# 7F2 Preosteoblast Cell Line Relative Viability Percentage after the Administration of 1% Roselle Flower Extract Nanoemulsion ( *Hibiscus sabdariffa* Linn.)

**DOI:** 10.1055/s-0045-1806950

**Published:** 2025-05-01

**Authors:** Ridhofar Akbar Khusnul Abdillah, Nadya Rafika Amalia, Theresia Indah Budhy, Muhammad Luthfi, Rini Devijanti Ridwan, Devi Rianti, Taufan Bramantoro, Nastiti Faradilla Ramadhani, Ida Bagus Narmada, Putri Cahaya Situmorang, Khairul Anuar Shariff, Tengku Natasha Eleena binti Tengku Ahmad Noor, Alexander Patera Nugraha

**Affiliations:** 1 Dental Health Science Study Program, Faculty of Dental Medicine, Universitas Airlangga, Surabaya, Indonesia; 2 Department of Oral and Maxillofacial Pathology, Faculty of Dental Medicine, Universitas Airlangga, Surabaya, Indonesia; 3 Department of Oral Biology, Faculty of Dental Medicine, Universitas Airlangga, Surabaya, Indonesia; 4 Department of Dental Material, Faculty of Dental Medicine, Universitas Airlangga, Surabaya, Indonesia; 5 Department of Dental Public Health, Faculty of Dental Medicine, Universitas Airlangga, Surabaya, Indonesia; 6 Department of Dentomaxillofacial Radiology, Faculty of Dental Medicine, Universitas Airlangga, Surabaya, Indonesia; 7 Department of Orthodontics, Faculty of Dental Medicine, Universitas Airlangga, Surabaya, Indonesia; 8 Study Program of Biology, Faculty of Mathematics and Natural Sciences, Universitas Sumatera Utara, Medan, Indonesia; 9 Department of Materials Engineering, School of Materials and Mineral Resources Engineering, Engineering Campus, Universiti Sains Malaysia, Penang, Malaysia; 10 Dentistry Division, 609 Armed Force Dental Clinic, Sarawak, Malaysia

**Keywords:** *Hibiscus sabdariffa*, preosteoblast, medicine, orthodontics, viability

## Abstract

**Objective:**

The roselle flower ( *Hibiscus sabdariffa* ) has shown potential as an alternative therapy for bone regeneration. This flower extract can induce osteoblast maturation, which is crucial for forming new bone. The study aim was to evaluate the viability of the 7F2 preosteoblast cell line following the application of a roselle flower nanoemulsion extract (RNE).

**Materials and Methods:**

This study utilized the 7F2 preosteoblast cell line to assess cell viability (%). The RNE was oven-dried at 35 to 40°C for 6 hours, resulting in a solid extract. The extract was then diluted into different concentrations. The preparation of 1% RNE was stirred at 1,400 rpm at 50°C for 90 minutes. Primary cultures of preosteoblast cell lines (7F2 cells) were distributed across 10 wells. Well 1 served as the positive control, representing 100% cell viability. Well 2 acted as the media control, containing only culture media without cells, representing 0% cell viability. Wells 3 to 10 were exposed to 1% RNE at serial concentrations of 100, 50, 25, 12.5, 6.25, 3.125, 1.56, and 0.78%. The viability of the 7F2 preosteoblast cell line was assessed using the 3-[4,5-dimethylthiazol-2-yl]-2,5-diphenyl tetrazolium bromide or microtetrazolium assay. The treatment was conducted on days 1, 3, and 7 for observation.

**Results:**

The findings indicated that the highest cell viability was observed on day 7, averaging 89.27% at a 0.78% concentration, while the lowest viability was 2.60% at a 100% concentration.

**Conclusion:**

These results suggest that RNE is nontoxic to the 7F2 preosteoblast cell line.

## Introduction

Hematopoietic and immunological cells in the bone marrow work together in the intricate physiological process of fracture repair. Growth factors, inflammatory cytokines, antioxidants, osteoclast and osteoblast cells, hormones, amino acids, and unidentified nutrients are among the several variables at play. 1 2 By offering three-dimensional frameworks for cell attachment, proliferation, and differentiation, bone tissue engineering is a potential technique for repairing bone tissue injuries that are difficult to mend. 3

Approximately 80% of people in underdeveloped nations still think that they receive primary medical care from traditional medicine, which is mostly based on plants and animals. Herbal remedies are in high demand and are becoming more and more popular each day. Because of their efficacy, affordability, and lack of adverse effects, herbal medications are generally favored. 4 Herbal medicine is said to hasten bone production without causing any negative effects and at a comparatively modest cost. 5 Numerous plant-based medications have been identified and utilized for bone repair. 6

Numerous biomolecules found in plant flowers, including alkaloids, glycosides, polyphenols, steroids, vitamins, terpenes, and others, have the potential to be used as therapeutic agents for treating bone disorders. A safe, affordable, and efficient alternative treatment for bone healing is the use of various plants and flowers in traditional therapies. 7 Previous studies have shown that the butanolic fraction and ethanolic extract from *Musa paradisiaca* flowers have antiresorptive and osteogenic properties, giving them an edge over parathyroid hormone, which is both bone-catabolic and bone-formation-promoting effects. 8

Bone defects naturally heal, but plants contain substances that may promote bone repair and prevent bone loss. Inadequate or delayed bone regeneration is a significant therapeutic concern. Certain plant constituents may downregulate biomarkers such as interleukin (IL)-1β, IL-6, IL-8, tumor necrosis factor (TNF)-α, and metalloproteinases 2 and 3, potentially increasing osseointegration. Additionally, they might enhance mediators such as type 1 collagen, osteocalcin, osteopontin, transforming growth factor-β1, vascular endothelial growth factor, and bone morphogenetic protein-2. Controlling the production of these cytokines could aid in bone repair.

Curcumol, caffeic acid, resveratrol, luteolin, and many other plant components may also be beneficial for bone health because they may inhibit osteoclast-mediated bone resorption, modulate Ca ^2+^ signaling and inflammatory mediator genes, and interfere with nuclear factor-κB (NF-κB) and mitogen-activated protein kinases. 9 Nonetheless, a wide variety of treatments employing herbal items, medications, and isolated metabolites are available. Therefore, using natural medicines requires greater specificity and precision, including the extraction and purification of individual components or molecules. 10

In recent years, natural plant-derived products have been extensively used as alternative therapies for bone regeneration and anti-inflammatory and antibacterial purposes due to their minimal side effects. 11 Indonesia, home to one of the largest tropical rainforests in the world, is located near the equator and hosts a diverse range of exotic herbs and medicinal plants, including the roselle flower ( *Hibiscus sabdariffa* Linn.). The roselle flower contains delphinidin-3-O-sambubioside (DOS), a compound that reduces the production of inflammatory mediators responsible for bone resorption. 12

A previous study found that without significantly reducing the expression of the surface marker for hematopoietic stem cells, the roselle flower exhibits cell-genoprotective properties during culture. It regulates the proliferation and intracellular antioxidant system of mouse bone marrow and hematopoietic stem/progenitor cells (Sca-1 ^+^ ), potentially through increased antioxidant capacity. According to these findings, hematopoietic stem/progenitor cells and cultured murine bone marrow cells react to foreign stimuli during proliferation, while roselle antioxidant flavonoids help replenish their intracellular antioxidant system. 13

Despite being a natural product, the roselle flower can still influence cell and tissue responses. Assessing these responses is crucial for evaluating the success of alternative biomaterials. *In vitro* viability tests on various cells, such as preosteoblasts (7F2 cell line), help determine bioavailability and the minimum dose that induces cell death. 14 Additionally, roselle flower extract at concentrations of 5 and 10% has demonstrated a reduction in alveolar bone damage in *Mus musculus* rats subjected to ligation on the second molar of the upper jaw. The treatment was applied both before and after ligation to assess its preventive and therapeutic effects. The findings indicated that roselle flower extract effectively inhibits alveolar bone resorption in rats following treatment. 15 However, studies on roselle flower nanoemulsion extract (RNE) remain limited. Therefore, this study aims to examine the impact of a 1% RNE on the viability of the 7F2 preosteoblast cell line *in vitro* . The objective was to assess the potential cytotoxicity of this biomaterial, facilitating its development for bone regeneration treatment.

## Materials and Methods

### Preparation of Roselle Flower Extract ( *H. sabdariffa* )

Roselle flowers were purchased from a local store in Malang, East Java, Indonesia. Fresh roselle flowers that showed no signs of physical damage or disease were picked, placed in dark plastic bags, and kept in a cool box. The samples were then prepared at the Dental Research Centre, Faculty of Dental Medicine, Universitas Airlangga.

After washing the leaves under running water to remove any contaminants, they were dried with a tissue. The extraction protocol for roselle flowers has been documented elsewhere. 15 Briefly, sun-dried roselle flowers were maintained at 50°C for 6 hours until the moisture content reached 8%. They were then sorted to remove damaged parts, crushed using a blender, and filtered through a 100-mesh sieve.

The resulting powder was transferred to a 3-L sealed jar and mixed with 2.5 L of 96% ethanol (at a 1:2 w/v ratio) as a maceration solvent and then allowed to stand for 3 days. The filtrate was stirred and separated from the residue through filtration. The total filtrate volume was 1,700 mL and was concentrated using a vacuum rotary evaporator (40–45°C; 6 hours). The roselle flower extract was then oven-dried (35–40°C; 6 hours), resulting in a solid extract weighing 128.7 g. 16 Additionally, the extract was diluted into different concentrations: 100, 50, 25, 12.5, 6.25, 3.125, 1.56, and 0.78%.

### Preparation of 1% Roselle Flower ( *H. sabdariffa* ) Nanoemulsion Extract

This preparation begins with heating distilled water to a temperature of 50 to 70°C in a glass beaker. Carboxymethyl cellulose sodium (CMC-Na) 1% gel and 1 g of Nipagin were weighed and placed in another beaker. Roselle flower extract ( *H. sabdariffa* ) was then mixed with 20 mL of 96% ethanol, stirred at 200 rpm in a beaker, and allowed to stand.

CMC-Na powder (1%) was then sprayed and stirred evenly in a mortar containing hot distilled water (45°C) and left for 15 minutes. Nipagin powder (purity 99%, cat. no. H3647, Sigma-Aldrich, United States) was dissolved in 10 mL of distilled water, stirred, and then mixed with 1% CMC-Na until homogenized. The roselle flower extract dissolved in 96% ethanol was poured into the mortar containing 1% CMC-Na and stirred at 200 rpm until homogeneous.

A Turrax machine (16,000 rpm for 10 minutes) was then used to mix the 1% nanoemulsion of roselle flower extract ( *H. sabdariffa* ) until a homogeneous mixture was obtained. The preparation was further stirred at 1,400 rpm at 50°C for 90 minutes. Thereafter, 5 mL of 1% (w/v) RNE was weighed for nanoemulsion testing and stored in a sealed, sterile container. The nanoemulsion procedure and particle size analysis followed the recommendations of a previously published report. The nanoemulsion of roselle flower ethanol extract was 96.13, showing that it had a good particle size and met the <300 nm nanoparticle criteria as mentioned in the previous study. 17

### Preparation of 7F2 Preosteoblast Cell Culture

Primary cultures of preosteoblast cell lines (7F2, CRL-12557, ATCC, United States) were obtained from stock at the Dental Research Center, Airlangga University, Surabaya, Indonesia. 7F2 cells were then maintained in Dulbecco's modified Eagle medium in 75 cm ^18^ conical tubes and allowed to grow until confluent. Cell cultures were incubated at 37°C in a humidified atmosphere of 5% CO _2_ , with the culture medium replaced every 48 to 72 hours. Cell attachment was performed by adding trypsin-ethylene diamine tetraacetic acid solution in phosphate-buffered saline. 18

The 7F2 preosteoblast cell culture was spread across 10 wells. Well 1 served as the positive control, representing 100% cell viability. Well 2 acted as the media control, containing only culture media without cells, representing 0% cell viability. Wells 3 to 10 were exposed to 1% RNE at serial concentrations of 100, 50, 25, 12.5, 6.25, 3.125, 1.56, and 0.78%. The microplate was incubated at 37°C for 24 hours before being removed from the incubator.

### Viability Calculation of Primary Culture of 7F2 Preosteoblast Cells

The viability of the 7F2 preosteoblast cell line was assessed using the 3-[4,5-dimethylthiazol-2-yl]-2,5-diphenyl tetrazolium bromide or microtetrazolium (MTT) assay. 7F2 preosteoblast cells (6 × 10 ^3^ cells/well) were added to a 96-well microplate. Following 24-hour incubation (37°C; 85–95% humidity; 5% CO _2_ ) (ESCO CelCulture Incubator 50 L, United States), cells were cultured for 3 to 4 days to reach 85 to 90% confluence.

The preosteoblast culture was then exposed to 20 μL of RNE at concentrations of 100, 50, 25, 12.5, 6.25, 3.125, 1.56, and 0.78%. Incubation was performed for 24 hours (37°C; 85–95% humidity; 5% CO _2_ ), followed by the addition of 10 μL of MTT (0.5 mg/mL). The plate was left static to allow formazan crystals to dissolve uniformly in each well and incubated at 37°C (85–95% humidity; 5% CO _2_ ) for 4 hours. 19

To maintain cell viability at a certain threshold, the media from each well was discarded, followed by the addition of 100 μL of dimethyl sulfoxide to dissolve the formazan salt. Absorbance in each well was measured using a microplate reader (Bio-Rad, model 550) at 490 nm. Each test was replicated four times independently. The treatment was performed on days 1, 3, and 7 for observation. This protocol followed the recommendations of previously published reports. 20 21

### Statistical Analysis

The data results were tested using the Kolmogorov–Smirnov's normality test ( *p* > 0.05), followed by a one-way analysis of variance parametric test ( *p* < 0.05). The test was then continued with the post hoc Tukey's test ( *p* < 0.05) to analyze the viability of 7F2 preosteoblast cells on days 1, 3, and 7 against each group and dose. Statistical analysis was performed using the Statistical Package for Social Science Software (SPSS) version 20.0 (IBM Corporation, United States).

## Results

### Viability of 7F2 Preosteoblast Cells after Administration of 1% RNE on Day 1

**Fig. 1:**
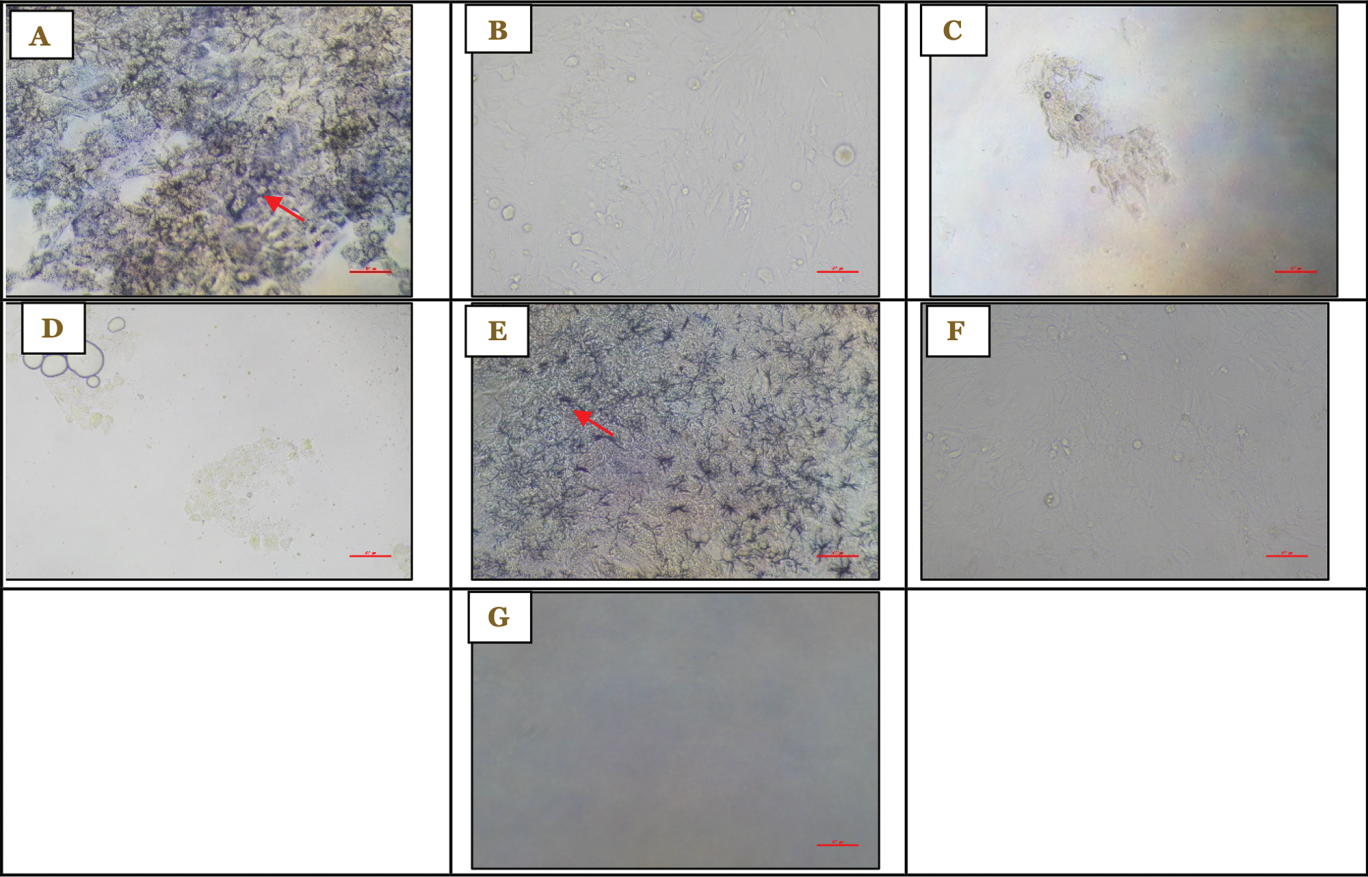
Viability of 7F2 preosteoblast cells after administration of 1% RNE at ×100 magnification at (A) 0.78% concentration after treatment, (B) 0.78% concentration before treatment, (C) 100% concentration after treatment, (D) 100% concentration before treatment, (E) control cells after treatment, (F) control cells before treatment, and (G) control medium as observed on day 1. RNE, roselle flower nanoemulsion extract. *Note: Formazan crystals are indicated by red arrows.*

**Fig. 2:**
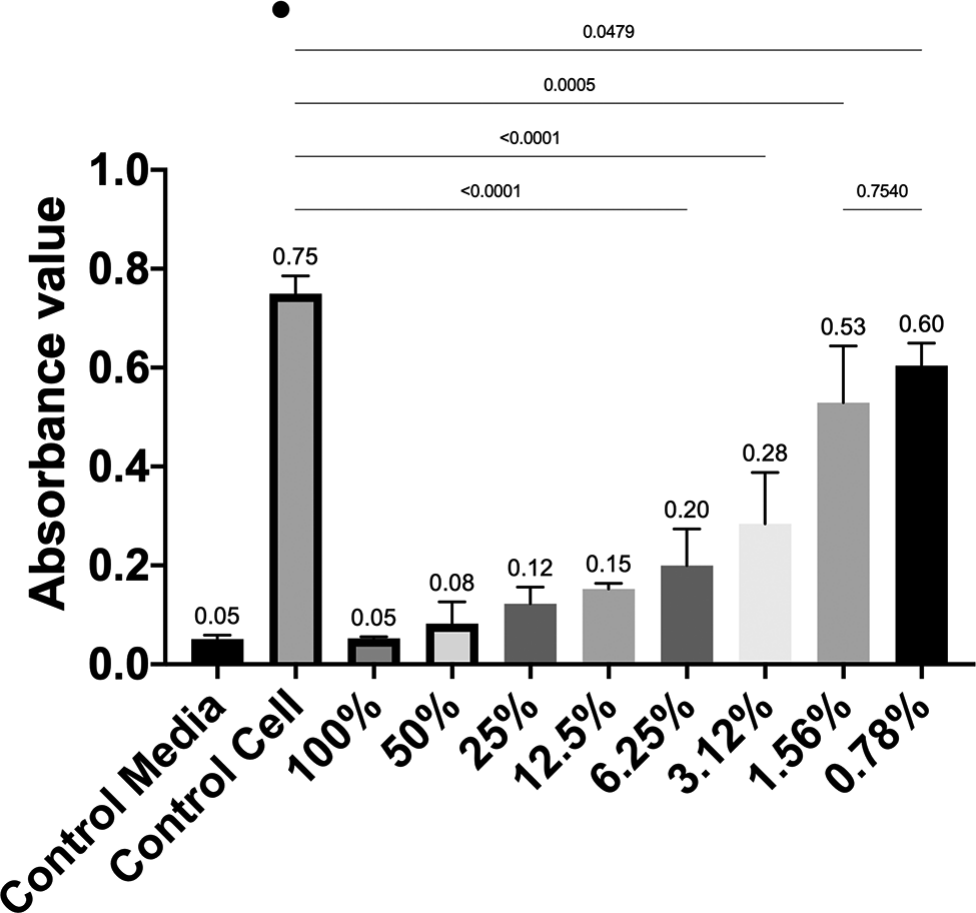
The absorbance value between observation concentrations among groups on day 1.

The RNE at 100% concentration had the lowest cell viability (0.25%) compared with the 0.78% concentration (79.14%), as shown by the crystal formazan formed in the 7F2 cells after treatment ( Table 1 ). The viability of 7F2 preosteoblast cells on day 1 is shown in Fig. 1 . The highest absorbance value on day 1 was at 0.78% concentration, while the lowest was at 100% concentration. There was a significant difference in absorbance values between the 0.78% concentration and the control cell group ( *p* < 0.05). However, there was no significant difference in absorbance values between the 0.78% concentration and the 1.56% concentration on day 1 ( *p* > 0.05) ( Fig. 2 ).

**Table 1:** Viability percentage of 7F2 preosteoblast cells as observed on days 1, 3, and 7 after application of 1% RNE

Observation	Cell viability (%)									
	Media control	Cell control	RNE concentration							
			100%	50%	25%	12.5%	6.25%	3.12%	1.56%	0.78%
Day 1	0	100	0.25	4.47	10.2	14.54	21.29	33.35	68.44	79.14
Day 3	0	100	0.649	7.71	8.16	12.64	19.26	37.75	73.48	88.55
Day 7	0	100	2.61	5.940	8.08	17.01	18.03	39.53	74.91	89.27

Abbreviation: RNE, roselle flower nanoemulsion extract.

### Viability of 7F2 Preosteoblast Cells after Administration of 1% RNE on Day 3

**Fig. 3:**
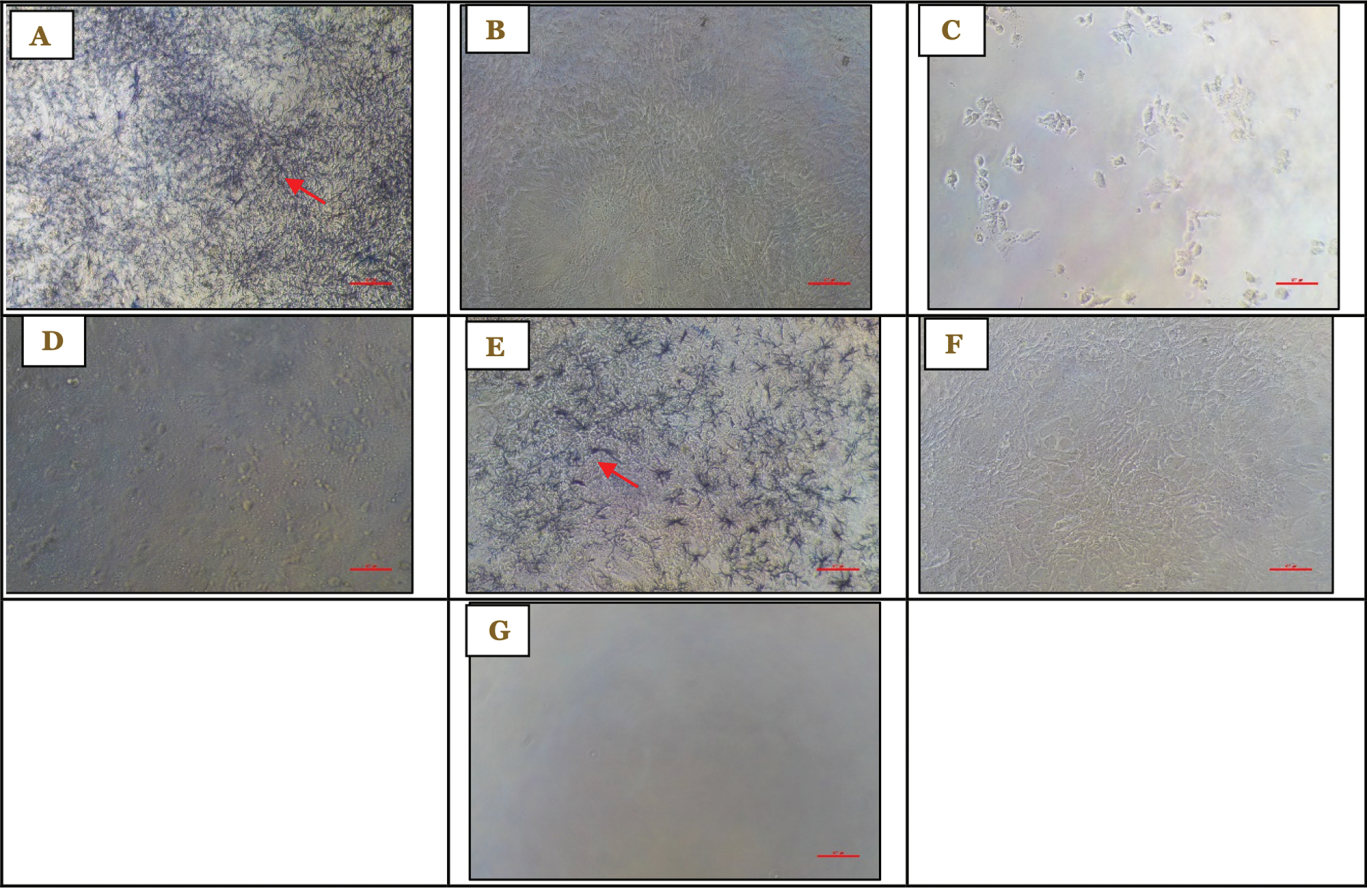
Viability of 7F2 preosteoblast cells after administration of 1% RNE at ×100 magnification at (A) 0.78% concentration after treatment, (B) 0.78% concentration before treatment, (C) 100% concentration after treatment, (D) 100% concentration before treatment, (E) control cells after treatment, (F) control cells before treatment, and (G) control medium as observed on day 3. RNE, roselle flower nanoemulsion extract. *Note: Formazan crystals are indicated by red arrows.*

**Fig. 4:**
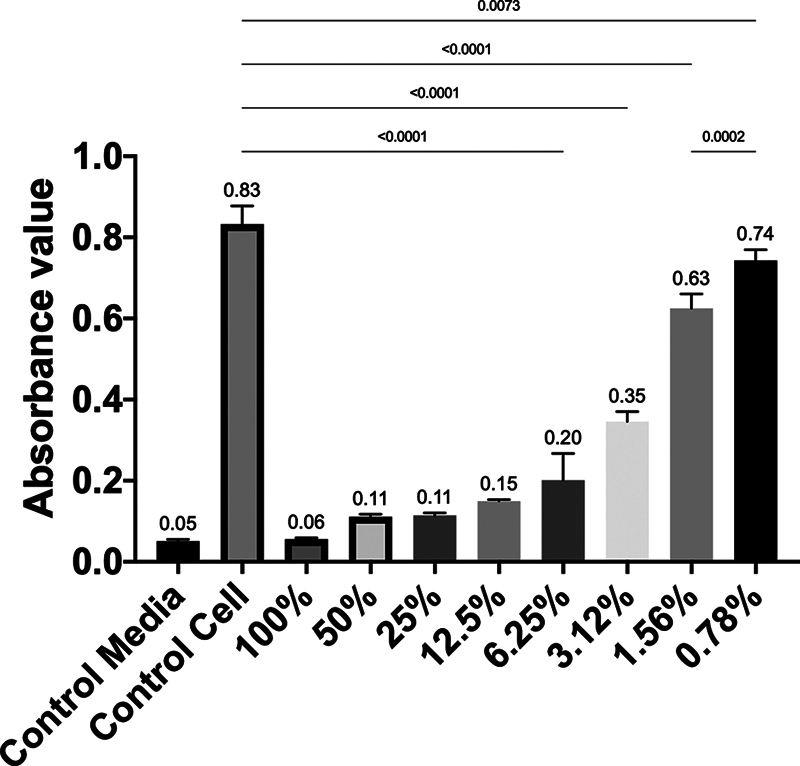
The absorbance value between observation concentrations among groups on day 3.

The RNE at 100% concentration had the lowest viability (0.639%) compared with the 0.78% concentration (88.547%), as shown by the crystal formazan formed in the 7F2 cells after treatment ( Fig. 3 ). The viability of 7F2 preosteoblast cells on day 3 is shown in Table 1 . The highest absorbance value on day 3 was at 0.78% concentration, while the lowest was at 100% concentration. There was a significant difference in absorbance values between the 0.78% concentration and the control cell group, as well as between the 0.78% concentration and the 1.56% concentration ( *p* < 0.05) ( Fig. 4 ).

### Viability of 7F2 Preosteoblast Cells after Administration of 1% RNE on Day 7

**Fig. 5:**
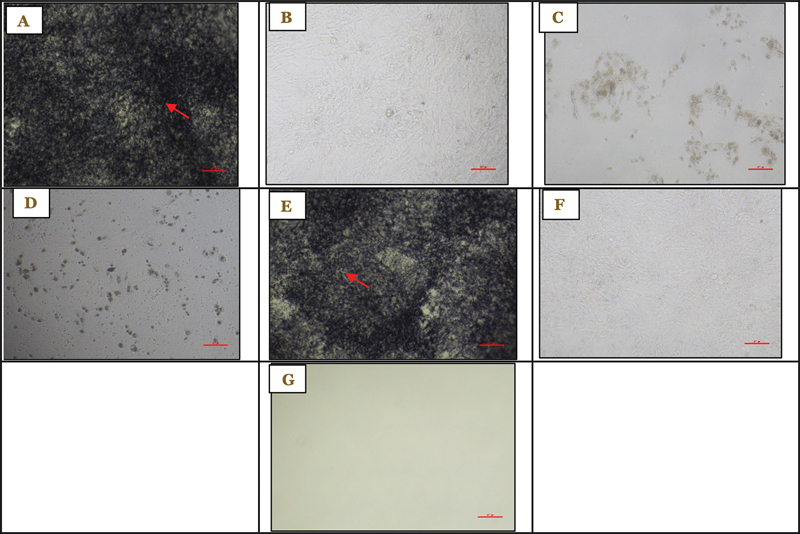
Viability of 7F2 preosteoblast cells after administration of 1% RNE under ×100 magnification at (A) 0.78% concentration after treatment, (B) 0.78% concentration before treatment, (C) 100% concentration after treatment, (D) 100% concentration before treatment, (E) control cells after treatment, (F) control cells before treatment, and (G) control medium as observed on day 7. RNE, roselle flower nanoemulsion extract. *Note: Formazan crystals are indicated by red arrows.*

**Fig. 6:**
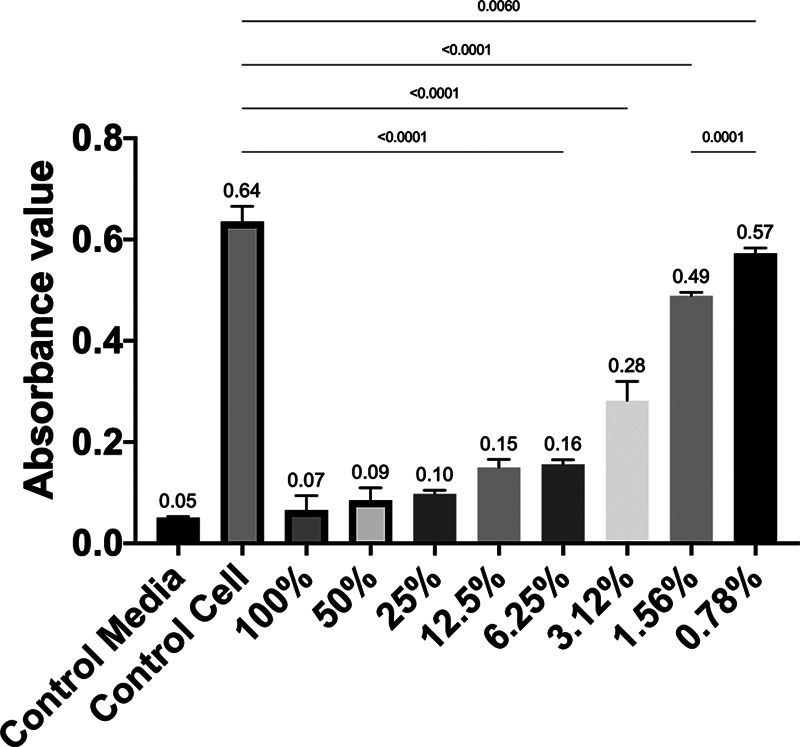
The absorbance value between observation concentrations among groups on day 7.

The RNE at 100% concentration had the lowest viability (2.606%) compared with the 0.78% concentration (89.273%), as shown by the crystal formazan formed in the 7F2 cells after treatment ( Table 1 ). The viability of 7F2 preosteoblast cells on day 7 is shown in Fig. 5 . The highest absorbance value on day 7 was at 0.78% concentration, while the lowest was at 100% concentration. There was a significant difference in absorbance values between the 0.78% concentration and the control cell group, as well as between the 0.78% concentration and the 1.56% concentration ( *p* < 0.05) ( Fig. 6 ).

## Discussion

In this study, we found that 1% RNE affects the viability percentage and absorbance value of the 7F2 preosteoblast cell line using the MTT assay at certain concentrations and observation days 1, 3, and 7. The highest absorbance value on days 1, 3, and 7 was at 0.78% concentration, while the lowest absorbance value was at 100% concentration. On the other hand, the lowest viability percentage of the 7F2 preosteoblast cell line was observed at 100% concentration on days 1, 3, and 7.

This finding is in line with a previous study, which proved that RNE at the smallest concentration of 1.96 ppm can stimulate the proliferation of fibroblast cells more (91.4%) than the highest concentration of 1,000 ppm (89.7%), leading to a decrease in cell viability. 22 According to previous studies, the lowest cell viability usually occurs on days 1 and 2, known as the lag phase, because cells need time to adapt to a new culture environment, attach, and begin spreading on the surface of the culture. As a result, cell viability may not increase significantly during this phase. This shows that increasing concentration will cause a decrease in the viability of 7F2 preosteoblast cells in each treatment but will increase as the days progress. 23

DOS and cyanidin-3-O-sambubioside (COS), polyphenolic chemicals found in RNE, have been shown in another study to activate macrophages and stimulate a variety of cytokines and enzymes. Both COS and DOS have anthelmintic, antiviral, antibacterial, and antioxidant properties. Anthocyanins increase antioxidant activity by decreasing plasma oxidation, thus lowering the rate at which fats and proteins oxidize. 24 RNE containing COS and DOS was administered to the culture of 7F2 preosteoblast cells to proliferate, increasing in number over time.

Through a paracrine effect, the 7F2 preosteoblast cell line released and induced cytokines and chemokines to proliferate, and then bone growth factors such as bone morphogenic protein-2 and alkaline phosphatase were secreted. 25 Bone growth factors then induced 7F2 preosteoblast cells to proliferate more on days 3 to 7, reaching a peak so that the viability of 7F2 preosteoblast cells increased. 26 27

The higher concentration of 1% RNE causes a decrease in preosteoblast cell viability when compared between groups on each day because, as the concentration of a compound increases, the aminoglycoside group also increases and may potentially make the compound toxic. The mechanism of cell death occurs through the diffusion process of glycoside groups through the pores in the cell membrane, causing cells to die due to the high levels of aminoglycoside groups as the concentration increases. The glycosidic group diffuses directly to the outer membrane of 7F2 preosteoblast cells, resulting in relatively high levels of cells and uncontrolled migration. Dead cells absorb the blue color of the trypan blue reagent and appear darker under the microscope. 20 21 23

While the living cells indicate that there is no more cell death because the lower concentration used contains relatively low glycoside groups. The glycoside group diffuses to the outer membrane of the tissue so that the 7F2 preosteoblast cells migrate slowly. The slow migration of cells stimulates the formation of cell wall substances to stay alive and proliferate so that metabolic changes in cells remain stable. The process of stable metabolic changes that do not occur substantially allows cells to remain alive longer. 28

Polyphenolic compounds that contain many glycoside groups in very high amounts as the concentration increases may be toxic, causing a decrease in cell viability. This is the main basis for determining the minimum level of cytotoxicity based on the concentration chosen, highlighting the need to identify the minimum effective concentration. 28 29

Roselle flower is an herbal-based biomaterial of natural origin containing polyphenolic compounds that have low side effects. 30 Roselle flowers are used in the preparation of 1% RNE to achieve good penetration due to their small particle size, which enhances the pharmacokinetics of drugs in tissues and reduces oxidative stress. 31 High oxidative stress in tissues causes inflammation, one of which is alveolar bone resorption. 32 High oxidative stress activates the nuclear factor kappa (NF-κB -light-chain-enhancer of activated B cells) pathway, which plays an important role in regulating inflammatory responses and osteoclastogenesis. Activation of NF-κB can increase the expression of receptor activator of NF-κB ligand, stimulating osteoclast differentiation and activity. The increase in both cytokines in the tissue leads to excessive bone resorption. 33 34 35

Oxidative stress can also increase the production of inflammatory cytokines, such as IL-6 and TNF-α, which inhibit osteoblast formation. 36 One percent RNE contains polyphenolic compounds in the form of DOS, which can reduce inflammation by inhibiting the activity of enzymes and molecules involved in the inflammatory process, such as cyclooxygenase and NF-κB, allowing bone regeneration to occur. The DOS compound can reduce proinflammatory cytokines IL-6 and TNF-α through the secretion of anti-inflammatory cytokines, such as IL-10, thereby reducing the inflammatory response and excessive bone resorption in the tissue. 24 30

The application of 1% RNE may stimulate osteoblastogenesis and the preosteoblast maturation process by increasing the migration of mesenchymal stem cells in inflamed tissue so that they can differentiate into preosteoblast cells. Preosteoblast cells will then dedifferentiate to produce osteoblasts in the bone matrix, and bone remodeling occurs. 15 30

Nevertheless, a drawback of this study is that it solely looks at the percentage of 7F2 preosteoblast cell line viability that RNE is biocompatible with, and it did not calculate the exact cell count. RNE biocompatibility with other osteoblast cell lines or in animal models of bone defects requires further research using various molecular methods such as mRNA fold examination, immunohistochemistry, signaling pathways, cytokine and growth factor levels, and clinical investigation methods such as micro-computed tomography scanner.

## Conclusion

Viability tests were conducted using 7F2 preosteoblast cells given RNE, with the highest relative viability at a concentration of 0.78% and the lowest at 100%. The statistical significance suggests that the decrease in relative viability was dose-dependent. Further studies are warranted to evaluate and investigate RNE administration in osteolysis *in vivo* using various biomolecular, cellular, and clinical investigation methods.
